# The Effects of Mechanical Scale on Neural Control and the Regulation of Joint Stability

**DOI:** 10.3390/ijms22042018

**Published:** 2021-02-18

**Authors:** Gil Serrancolí, Cristiano Alessandro, Matthew C. Tresch

**Affiliations:** 1 Department of Mechanical Engineering, Universitat Politècnica de Catalunya, 08019 Barcelona, Spain; 2 Department of Brain and Behavioral Sciences, Università degli Studi di Pavia, 27100 Pavia, Italy; cri.alessandro@gmail.com; 3 Department of Physiology, Northwestern University, Chicago, IL 60611, USA; m-tresch@northwestern.edu; 4 Department of Biomedical Engineering, McCormick School of Engineering, Northwestern University, Evanston, IL 60208, USA; 5 Department of Physical Medicine and Rehabilitation, Northwestern University, Chicago, IL 60611, USA; 6 Shirley Ryan AbilityLab, Chicago, IL 60611, USA

**Keywords:** joint stability, ligament, mechanical scale

## Abstract

Recent work has demonstrated how the size of an animal can affect neural control strategies, showing that passive viscoelastic limb properties have a significant role in determining limb movements in small animals but are less important in large animals. We extend that work to consider effects of mechanical scaling on the maintenance of joint integrity; i.e., the prevention of aberrant contact forces within joints that might lead to joint dislocation or cartilage degradation. We first performed a literature review to evaluate how properties of ligaments responsible for joint integrity scale with animal size. Although we found that the cross-sectional area of the anterior cruciate ligament generally scaled with animal size, as expected, the effects of scale on the ligament’s mechanical properties were less clear, suggesting potential adaptations in passive contributions to the maintenance of joint integrity across species. We then analyzed how the neural control of joint stability is altered by body scale. We show how neural control strategies change across mechanical scales, how this scaling is affected by passive muscle properties and the cost function used to specify muscle activations, and the consequences of scaling on internal joint contact forces. This work provides insights into how scale affects the regulation of joint integrity by both passive and active processes and provides directions for studies examining how this regulation might be accomplished by neural systems.

## 1. Introduction

Central pattern generators (CPGs) for rhythmic locomotor outputs have been demonstrated in vertebrates, from zebrafish to humans [1,2,3,4]. Many aspects of these CPGs appear to be similar across vertebrates, including molecular and genetic profiles of spinal interneurons and their developmental progression [3,4,5,6,7]. In addition to the common evolutionary history shared by vertebrates, this similarity is consistent with the often similar musculoskeletal structures shared across vertebrates, suggesting that the necessary neural control strategies for producing behavior might also be similar. For example, the hindlimb of mice and the leg of humans contain many of the same basic musculoskeletal elements, including specific muscles and bones, although there are obvious specializations according to each organism’s behavioral repertoire and evolutionary niche.

Even though limb structure might be similar across many vertebrates, the different size of each animal will significantly alter the mechanical properties of their musculoskeletal systems, potentially altering the control problem when producing behavior. Several analyses have highlighted how mechanical properties of the musculoskeletal system are altered across scales [8,9]. For example, if the length of a bone doubles (i.e., the length scale factor is 2, $s_{l}=2$) and all other aspects of the bone remain unaltered (e.g., density, material properties, shape), then its mass increases by a factor of 8 (i.e., the mass scale factor is 8, $s_{m}=8$, and more generally, $s_{m}=s_{l}^{3}$). Or equivalently, if the mass doubles ($s_{m}=2$), then the length of the organism should increase by 2^1/3^ ($s_{l}=2^{1/3}$) [8]. These scaling properties imply that mass and inertia become increasingly significant as animals become increasingly large.

The consequences of these mechanical scaling properties have been investigated in many studies and previous reviews [8,9,10], showing how animals’ posture, behavioral speed, and hindlimb excursion angle, among other mechanical variables, are altered across organisms of different sizes. However, few studies have investigated how neural strategies for motor coordination change across animal scales. Hooper et al. [11,12] considered the effects of scaling on the control of limb movement and the contributions of passive elastic structures. As described above, mass and inertial properties scale with the cube of the length scale (or, equivalently, length scale with the cube root of mass). At the same time, forces due to passive limb structures, such as ligaments, tendons, or passive elastic elements within muscles, should scale with the square of the length scale since the forces due to these structures are generally related to their cross-sectional area (CSA), e.g., the amount of force generated by a stretched ligament will be related to the number of stretched collagen fibers in the ligament and so the amount of force produced by a ligament should generally be proportional to its CSA. Thus, forces due to inertial properties will increase with body size faster than forces due to passive elastic properties, suggesting that passive elastic elements should play a significant role in motor control for small animals, whereas inertial properties should play a significant role in larger animals. As described in Hooper et al., these scaling properties have consequences for neural strategies underlying motor control, influencing muscle activations during both posture and movement.

Limb posture and trajectories are obviously critical when considering animals’ task performance and ability to achieve behavioral goals. However, recent experiments [13,14,15] have demonstrated the importance of another set of variables to the neural control of movement, those characterizing the state of internal joint structures. Although the regulation of joint integrity has long been studied in sports medicine and clinical biomechanics, the importance of this issue in understanding neural control strategies has not been well appreciated. Our recent research [13,14,15] has established that the coordination patterns of muscle activations and their adaptation following perturbations or injuries reflects the regulation of stresses within joints, such that net joint stresses are reduced while maintaining limb posture and trajectories so that task performance is achieved. Other recent work has demonstrated the critical role of neural systems in the proper development of joints [16,17,18,19].

Although that work establishes that the nervous system plays an important role in the regulation of joint integrity, passive structures within joints such as ligaments or capsular membranes are also critical in this regulation. The same analyses described by Hooper et al. might therefore be expected to apply in this situation as well: the forces that destabilize a joint due to mass/inertia should scale with the cube of the length scale of an animal, whereas the passive forces that resist these destabilizing forces should scale with the square of the length scale. This idea suggests that, in the absence of other adaptations, passive joint elements should be less able to stabilize joints in larger animals and so neural control will become more critical.

In this article, we consider these issues related to the passive and active regulation of joint stabilization. We first perform a literature review, considering how the anatomical and mechanical properties of ligaments involved in joint stabilization scale with animal size. We evaluate whether the scaling of these properties is determined solely by animal size, or if there are compensatory adaptations in ligament properties specific to each animal to ensure joint stabilization across scales. We then consider how changes in animal scale affect the neural control strategies for maintaining joint stability in the face of perturbations. We perform a series of simulations evaluating how the muscle activations required to maintain limb configuration in response to perturbations are altered with animal size. We demonstrate the importance of the cost function used to resolve muscle redundancy in determining the scaling of muscle activations with animal size and show how these changes in muscle activation affect the internal joint forces that might compromise joint integrity. These analyses and literature reviews provide new perspectives on how joint stabilization is affected by changes in animal size, highlighting the importance of animal scale in the role of both passive limb structures and neural control strategies.

## 2. Scale-Dependent Contributions of Passive Structures to Joint Stabilization

Several structures within joints have passive properties that can help stabilize a joint against tangential or normal contact forces in order to maintain joint integrity. Ligaments are the main structures involved in preventing joint dislocation from aberrant tangential forces. For example, in the mammalian knee joint, the anterior cruciate ligament (ACL) prevents anterior translation of the tibia relative to the femur. Rupture of the ACL is a common knee joint injury [20], suggesting that this ligament plays an important role in joint stabilization [21]. Because of its clinical relevance and its importance across mammals [22], we performed a literature review examining how anatomical and mechanical properties of the ACL scale with organism size, evaluating how the scaling principles described above are reflected in ACL properties.

We searched for experimental values of the CSA of the ACL and body mass from cadaveric specimens of different species. Three studies were found reporting data for rabbits [23,24,25], one for rhesus monkeys [26], one for goats [27], one for sheep [28], one for three disaggregated specimens of ponies [29], and one for cows [30]. Two studies reported values for humans (four data groups) [26,31].

**Figure 1:**
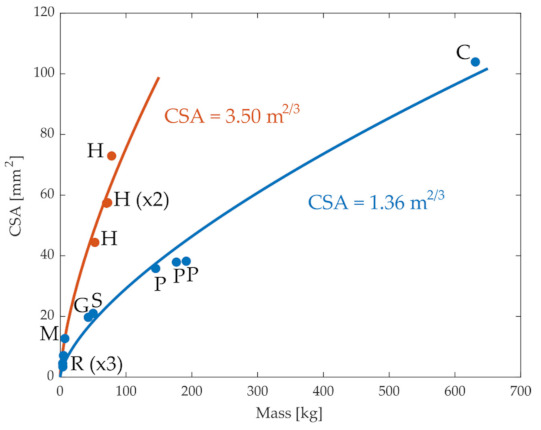
Values of the cross-sectional area (CSA) of knee anterior cruciate ligament for different species from the literature. C: cow, G: goat, H: human, M: monkey, P: pony, R: rabbit, S: sheep. In red, human values (bipeds), and in blue, quadrupeds. Blue and orange lines are the best-fit least-square regressions for humans and quadrupeds.

Figure 1 illustrates the relation between the CSA of the ACL in different species and their mass. In general, the CSA increased with organism size, as expected, although the data from humans appeared to deviate from that of other mammals. We evaluated whether this increase followed the expected relation between CSA and mass. Considering that CSA depends on the square of a length measurement, and in turn, the length scales with the mass to the power of 1/3, this relationship can be written in the form of $CSA=a\;m^{2/3}$. The parameter *a* can be interpreted as a ratio $CSA_{0}/m_{0}^{2/3}$ for some reference data pair to which all data points are scaled; *a* was chosen as the least squares fit to the observed data. As shown by the blue line in Figure 1, this equation described the relationship between CSA and mass in non-human mammals very well (r = 0.99). Similarly, when data from only humans were considered, the equation fit the data well (r = 0.92). However, when the data were combined from both humans and non-humans, the quality of the fit was substantially reduced (r = 0.81), suggesting that this relationship between CSA and mass is not consistent across all animals. One possibility is that the deviations between humans and the other mammals reflect an adaptation in ACL CSA due to the bipedal gait of humans and the larger proportion of body mass accommodated by each knee [32]. Indeed, when the fit was repeated but considering the mass divided by the number of weight-supporting limbs, the quality of fit improved (r = 0.92). These observations suggest that the expected relationship between ACL geometry and body mass ($CSA=a\;m^{2/3}$) holds as expected based on geometric scaling for most quadrupedal mammals, but there are apparent adaptations in humans, potentially reflecting humans’ bipedal gait.

We next evaluated whether these alterations in ACL geometry resulted in the expected alterations in ACL mechanical properties. If the material properties of the ACL remained constant across animals, one would expect that the stiffness of the ACL would scale consistently with its dimensions [33]. In the linear region of the ligament, the force $F_{ACL}$ is linearly proportional to ligament elongation Δl, and the slope of this relationship represents the stiffness *k* of the ligament: $F_{ACL}=k\Delta l$. Considering that the maximum force of the ACL scales with the CSA, as mentioned above, the scaling factors of stiffness, length, and mass should be regulated by the following relationship:

(1) $$s_{F_{ACL}}=s_{k}s_{l}=s_{k}s_{m}^{1/3}=s_{m}^{2/3}$$

which implies that $s_{k}=s_{m}^{1/3}$; where $s_{F_{ACL}}$ is the scale factor of the ACL force and $s_{k}$ is the scale factor of the stiffness. We therefore evaluated this relationship between the scaling properties of mass and ACL stiffness against experimental data from the literature.

**Figure 2:**
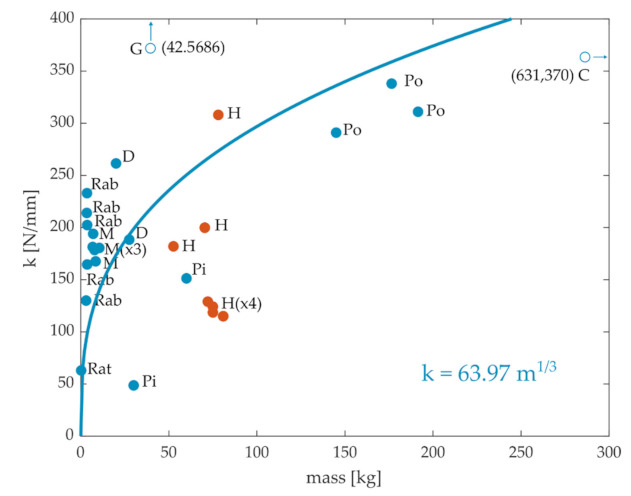
Stiffness of the anterior cruciate ligament (ACL) versus mass for different species obtained from the literature. C: cows, D: dogs, G: goats, H: humans, M: monkeys, Pi: pigs, Po: ponies, Rab: rabbits. There are two outliers in the plot, the stiffness values of the goats and the cows. The blue line is the best-fit least-square regression for the quadrupeds.

A literature review of ACL stiffness and mass values from cadaveric specimens of different species was performed. We obtained 27 data points, including rats [34], rabbits [25,35,36,37], monkeys [26,38,39,40], pigs [41,42], dogs [38,43], goats [27], ponies [29], cows [30], and humans [26,31,44,45], as illustrated in Figure 2. Although ACL stiffness generally increased with body mass across animals, there was not an obvious systematic relationship that held across all mammals. For example, the stiffness of rabbits and monkeys seems to be in the same order of magnitude as that of humans. Note also the much higher stiffness for goats. Moreover, the range of variation within the same species is quite high (see Rab and H). The quality of the fit between stiffness and mass using Equation (1), even when considering only quadrupeds, was considerably worse (r = 0.5) than that observed between CSA and mass.

**Figure 3:**
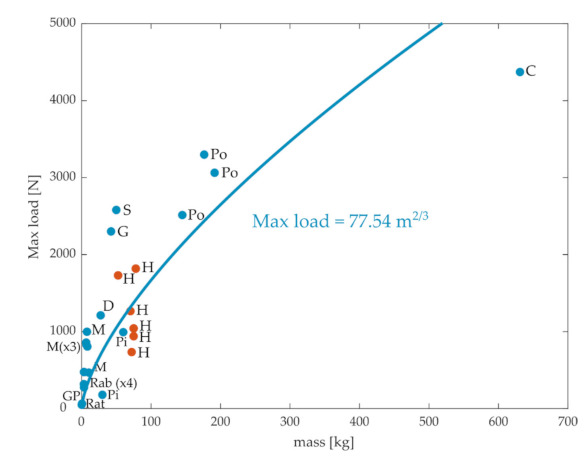
Maximum load before rupture of the ACL versus mass for different species obtained from the literature. C: cows, D: dogs, G: goats, GP: guinea pigs, H: humans, M: monkeys, Pi: pigs, Po: ponies, Rab: rabbits, S: sheep. The blue line is the best-fit least-square regression for the quadrupeds.

Although stiffness characterizes one aspect of how the ACL resists tibial translations, another clinically relevant characteristic of the ACL is the maximum load that it can support before rupture. If all intrinsic properties of the ACL were maintained across animal scales, the maximum load should be determined solely by the CSA of the ACL, and so should scale with the mass to the 2/3 power. Experimental values of the maximum ACL load obtained from the literature are plotted in Figure 3. We obtained 26 data points, including rats [34], rabbits [25,36,37], monkeys [26,38,39,40], pigs [41,42], guinea pigs [46], dogs [38], goats [27], sheep [28], ponies [29], cows [30], and humans [26,31,45]. Again, there was a general pattern, so maximum load increased with the animal’s mass, although there was a high degree of variation within species (e.g., humans) [31]. The fit between body mass and maximal force showed substantial deviations from the observed data, even though the quality of the fit for quadrupeds was reasonable (r = 0.89).

The relationships between ACL stiffness (Figure 2) or maximum load to rupture (Figure 3) and body mass thus do not obviously appear to follow those expected based on simple scaling laws. On the other hand, the relationship between CSA and body mass is more consistent with expected scaling principles, although there appeared to be some adaptations in humans reflecting their bipedal gait. These observations suggest that intrinsic mechanical properties of ligaments are adapted, at least to some extent, according to species specific behavioral requirements. There are potentially several sources that might explain these deviations from expected behavior. For instance, ACL stiffness has been shown to vary with sex and age [31,39,45,47], potentially reflecting differences in ACL material properties across populations in the same species. It has also been shown that the maximum load before ACL rupture depends on the orientation of the femur relative to the tibia. Rogers et al. [28] showed that the maximum load could be doubled when the stress–strain test was performed with a 45° knee flexion compared to the case where the knee had no flexion. This observation suggests that the maximum load for the ACL in animals with a crouched hindlimb posture might not need to be as high as that for animals with straight legs. Accordingly, closer inspection of the data in Figure 3 suggests that there might be a different relationship between maximum load and body mass in animals with crouched hindlimbs postures (rats, rabbits, monkeys, dogs) as opposed to animals with more straight legs (cows, ponies, sheep, goats). Finally, the differences in stiffness or maximal load might also reflect adaptations in ligament properties, such as fiber density [48,49] or anatomical organization [50,51], according to behavioral requirements for each species.

## 3. Effects of Scaling on the Neural Control of Joint Stabilization

In this section, we consider how neural control strategies for joint stabilization are affected by scaling principles. We consider two aspects of joint stabilization against unexpected perturbations [52,53]. The first aspect is the maintenance of limb configuration, ensuring that joint angles and overall posture are maintained so that behavioral goals are achieved. This regulation requires that joint moments imposed by external perturbations are countered by the moments produced by passive and active musculoskeletal elements. The second aspect concerns the tangential and normal contact forces between bones produced by the perturbation. Tangential forces between bones might lead to ligament strain or rupture whereas contact forces between bones might lead to cartilage degradation and osteoarthritis. Again, these forces can be countered by either active or passive elements in the limb [54].

How does the problem of maintaining joint stability change with the mechanical scale of an organism? We examined this issue using a simple biomechanical model of the rat hindlimb. We used an OpenSim model [55] of the rat hindlimb with 5 segments (spine, pelvis, femur, tibia, and foot) and 13 degrees of freedom (dofs) (3 rotations and 3 translations of the spine with respect to the ground, 3 dofs at the hip, 1 at the knee, and 3 at the ankle) (Johnson et al. [56]). The pose of the animal was fixed. We then applied an anterior–posterior perturbation of 5 cm in one second (with continuous velocities and accelerations) that caused a translation of the animal and changes in ground reaction forces during this translation. The OpenSim Scale Tool was used to obtain four models with body masses of 100, 200, 300, and 400 g. Inverse dynamics analyses were applied to these four models under the same perturbation and scaling the ground reaction forces according to the altered size of the model, in order to obtain the joint moments required to maintain the posture under the perturbation and the corresponding internal joint forces.

**Figure 4:**
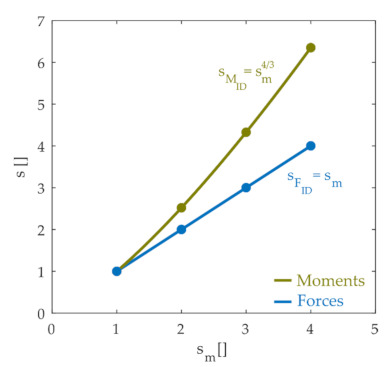
Scale factors of inverse dynamic forces (blue dots) and inverse dynamic moments (green dots) with respect to the scale factors of the mass. The line and the equations are fitted from the dots. These joint moments and forces are those that must be produced in order to oppose the perturbation and maintain joint configuration and joint integrity.

We then examined how the joint moments and internal contact forces scaled with the size of the animal. Figure 4 illustrates how inverse dynamics joint moments and forces scale with the mass of the animal, showing that the internal joint forces varied linearly with the mass scale factor whereas joint moments scaled with the mass scale raised to the 4/3 power. These scaling results are not surprising and are expected based on basic biomechanical principles. The linear scaling of force with mass is a straightforward consequence of Newton’s laws. Since lengths scale with the cube root of the mass scale factor ($s_{m}=s_{l}^{3}$; $s_{l}=s_{m}^{1/3}$) and forces scale linearly with mass, joint moments, which are the product of a force and distance, scale with the mass raised to the 4/3 power. The simulation results shown in Figure 4 therefore confirm these basic biomechanical principles. These forces and moments must be countered by a combination of active and passive elements in order to stabilize the limb and maintain joint integrity.

The simulation work described above demonstrates how aggregate limb mechanics at the level of joint moments or internal interaction forces scale with animal size. The nervous system, however, does not act on these aggregate limb mechanics directly but, instead, controls these variables indirectly by the activation of muscles. Both the force produced by a muscle and the muscle moment arms that determine how muscle force is translated into joint moments vary with the size of an animal. We therefore considered how scaling impacts neural control strategies for maintaining joint stability at the level of muscle activations.

We evaluated these issues using the rat hindlimb model described above (developed from [56]), creating musculoskeletal models for animals of different masses. This model has 38 muscles spanning the hip, knee, and ankle joints. We adjusted the properties of each muscle according to scaling principles. These adjustments assume no adaptations in intrinsic muscle function across scale, but only reflect geometric differences due to changes in animal size. Optimal fiber lengths and tendon slack lengths were scaled with the same scale factor as the length dimension ($s_{l_{s}^{T}}=s_{m}^{1/3}$ for tendon slack length and $s_{l_{0}^{M}}=s_{m}^{1/3}$ for optimal fiber length). The maximum isometric force for each muscle is related to the muscle’s physiological CSA, and so was scaled with a factor of $s_{F_{iso}^{\max}}=s_{m}^{2/3}$, as suggested by Van der Kroogt et al. [57]. We assumed that normalized active force–length ${\tilde{F}}_{l}^{M}$, active force–velocity ${\tilde{F}}_{v}^{M}$, and passive muscle force ${\tilde{f}}_{pe}$ relationships do not depend on scale. Passive muscle forces had to be slightly adjusted from the original model in order to avoid numerically infeasible passive force values; these adjustments were made in the original model, then preserved across the different scaled models. Muscle moment arms are determined by the muscle path within the skeleton and so scale with the same factor as the length dimension (i.e., $s_{ma}=s_{m}^{1/3}$).

Using these musculoskeletal models, we then considered how neural control strategies for joint stabilization change according to body size. In order to stabilize the joint configuration in response to a perturbation, the joint moments produced by muscles should be equal and opposite to the joint moments induced by the perturbation. As shown above, the perturbation-induced joint moments scale with animals’ size according to $s_{M_{ID}}=s_{m}^{4/3}$. Since the joint moments produced by muscles are given by $F^{T}\cdot ma$ (where $F^{T}$ is the force exerted by the tendon on the skeleton), the forces produced by each muscle–tendon unit (*F^T^*, from tendon force) should scale linearly with the mass of the animal: $s_{F^{T}}=s_{m}$. The dependence of muscle force on its activation was modeled according to the common relationship:

(2) $$F^{T}=\left(a{\tilde{F}}_{l}^{M}{\tilde{F}}_{v}^{M}+{\tilde{f}}_{pe}\right)F_{iso}^{\max}\cos\alpha$$

where *a* is the muscle activation (ranging between 0 and 1, with 1 indicating maximal activation), and *α* is the pennation angle of muscle fibers. Pennation angle was assumed to be near constant in this analysis since we are analyzing the quasi-static task of maintaining joint configuration. For simplicity, consider first the case in which there are no passive muscle forces:

(3) $$F^{T}=a{\tilde{F}}_{l}^{M}{\tilde{F}}_{v}^{M}F_{iso}^{\max}\cos\alpha$$

From the analyses described above, tendon force should scale linearly with animal mass in order to produce the joint moments necessary to maintain joint configuration. Since the maximum isometric force of a muscle ($F_{iso}^{\max}$ in Equation (3)) scales according to $s_{F_{iso}^{\max}}=s_{m}^{2/3}$, muscle activations should therefore scale according to $s_{a}=s_{m}^{1/3}$ in order to produce the joint moments necessary to counteract the imposed perturbations and maintain joint configuration.

This analysis demonstrates that neural control strategies for maintaining joint configurations must be altered according to the size of the animal, but it suggests a relatively simple scaling principle ($s_{a}=s_{m}^{1/3}$) for altering muscle activations. This principle is complicated, however, by two additional factors. First, passive muscle forces will alter the required scaling of muscle activations: if these passive forces assist in joint stabilization, the scaling of muscle activations across animal size should be lower than $s_{a}=s_{m}^{1/3}$, although the precise form of this scaling is difficult to predict from first principles. Second, the above analysis assumes that the relative distribution of forces across muscles remains invariant across animal size. However, because of muscle redundancy, this distribution will depend on the cost function that is optimized to choose the pattern of muscle activations. For instance, a cost function equal to the sum of muscle activations squared penalizes large activations more than small activations. Minimization of this cost function will therefore tend to cause activation of a larger number of muscles as overall activation levels increase, since it is less costly to activate many muscles at a low level than to activate one muscle at a high level. This effect will also tend to reduce the scale factor for muscle activations across body sizes.

**Figure 5:**
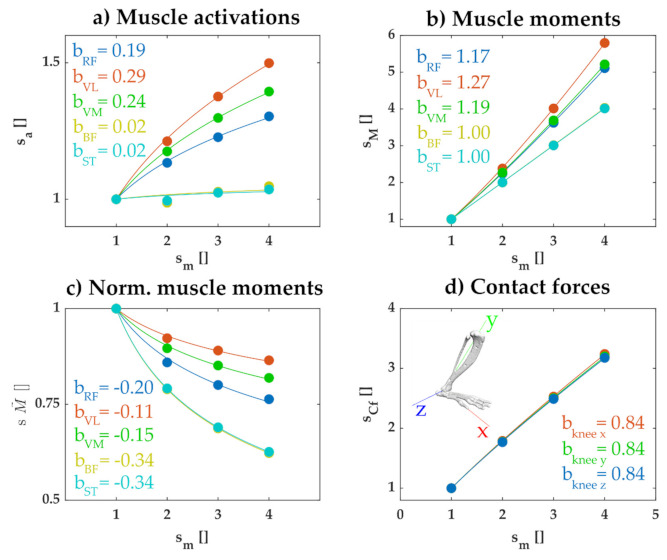
Scale factors obtained for rectus femoris (RF), vastus lateralis (VL), vastus medialis (CM), biceps femoris (BF), and semitendinosus (ST). These muscles are illustrated since they reflect the main muscles acting at the knee joint in these simulations. The scale factors are obtained by dividing the maximum values of the variables through the trial by the maximum value at the lowest scale. Note that all scale factors start with a value of 1. The variables analyzed are: (**a**) muscle activations, (**b**) knee joint moments produced by each muscle, (**c**) muscle contribution to the knee joint moment, calculated as a fraction of the total knee joint moment, (**d**) knee contact forces in x, y, and z directions. *b* is the exponent of the fitted expression $s_{x}=s_{m}^{b}$, where *s_x_* is the scale factor of the corresponding variable. Note that parameter *a* used for the fits in Figure 1, Figure 2 and Figure 3 is omitted, since it is equal to 1 in each plot here because all values are expressed relative to the simulation with body mass of 100 g.

The results of simulations confirmed these general considerations. We used the rat hindlimb model for each scale of animal and calculated the set of muscle activations that minimized the summed squared activations while maintaining joint configuration during the perturbation. Figure 5a illustrates how the activation for a subset of muscles increased with the mass of the animal. The activation of each muscle increased according to a scale factor less than $s_{m}^{1/3}$, with the exact scale factor differing for each muscle. In separate simulations, we confirmed that this lower than $s_{m}^{1/3}$ scaling factor reflected the effects of passive muscle forces and the cost function used; when we repeated the simulation without passive muscle forces and using a cost function with the summed activations (not squared), the activation of recruited muscles all scaled according to $s_{m}^{1/3}$ (not shown). Similarly, we observed that the joint moment produced by each muscle increased with mass with a factor less than the expected $s_{m}^{4/3}$ (Figure 5b) and the relative contribution of each muscle to the net joint moment decreased with increasing animal size (Figure 5c). These results reflect the expected wider distribution of muscle activations across multiple muscles as animal size increases, due to passive forces and the squared activation cost function.

In the analyses described above, muscle activations were chosen in order to maintain joint configuration, without consideration to their consequence on the internal tangential and normal contact forces within each joint. These muscle activations can affect these contact forces and these effects might vary according to the scale of the animal. We therefore examined how muscle activations chosen to maintain joint configuration affected the joint contact forces at the knee imposed by the perturbations. As illustrated in Figure 5d, the joint contact forces increased with body mass, but with an exponent less than 1. Thus, even though the joint forces imposed by the perturbations increased linearly with body mass (Figure 4), the joint contact forces produced when muscle activations were used to maintain joint configuration increased with body mass at a reduced, sublinear scale factor. 

This reduced scaling factor of contact forces, when muscles are activated to maintain joint configuration, was not a necessary result of any control strategy. When we repeated these simulations but used a cost function consisting of the summed activations and excluded passive forces, we found that net contact forces increased linearly with body mass. This latter observation is consistent with the analyses described above; since under these conditions the distribution of muscle forces is constant across scales and the net muscle force scales linearly with body mass, we therefore expect that the contact force due to muscles should also scale linearly with body mass. This observation suggests that the sublinear increase in joint contact forces observed in Figure 5d reflects the wider distribution of activated muscles observed when using a quadratic cost function and including passive muscle properties.

## 4. Conclusions

We examined several aspects of how the mechanical scale of an organism affects the regulation of joint stability, evaluating the scale-dependent properties of passive structures in joints and how neural control strategies for joint stabilization are altered across scales. We found that the scaling of anatomical and mechanical properties of the ACL did not generally have a clear relationship with body mass, suggesting the possibility that changes in ACL properties may be specific to each animal depending on their specific behavioral demands, in order to better ensure joint integrity. We further showed that the neural control strategy used to maintain joint configuration in response to perturbations must be significantly altered across animal scales, requiring activation of a larger number of muscles with increasing animal size. Although this result suggests that neural control strategies differ between animals of different scales, we also found that these alterations also caused internal joint contact forces to increase with animal size at a rate less than expected. We consider each of these observations below.

### 4.1. The Effects of Scaling on Passive Elements Involved in Joint Integrity

Based on a review of the literature examining properties of the ACL, we found that the CSA of the ACL in quadrupeds generally scaled to body mass, as expected, as did the CSA of the ACL in bipeds. However, a common scaling principle could only be identified across both groups by normalizing body mass by the number of weight-supporting limbs. This observation suggests that ACL geometry is not strictly determined by animal size but is adapted according to the altered joint contact forces produced in bipedal behaviors.

Although ACL mechanical properties (stiffness and maximum load) generally increased with increasing animal size, there was not a clear systematic relationship between these properties and body mass. The lack of a clear relationship for these properties might simply be due to variations in experimental methods across studies. For instance, measurements of stiffness can vary depending on the length of the ligament at which the stiffness is measured or on how ligament samples are prepared. Alternatively, the variations in these properties might reflect adaptations in ligament properties to species-specific behavioral demands, involving changes in material properties or internal configuration of the collagen fibers comprising ligaments. Future studies using identical experimental procedures to characterize ligament mechanical properties across animals or relating ligament properties to species-specific behaviors might help clarify these issues further to determine how ligament properties are altered across species.

### 4.2. The Effects of Scaling on the Neural Control of Joint Stabilization

The computational work described in Section 3 extends previous work evaluating how neural control strategies are altered across changes in body size [6,11]. One basic result of our simulations was to show that muscle activations must increase with increasing body size in order to maintain a joint configuration in the face of imposed perturbations. This result is a consequence of basic scaling principles described previously; since the muscle forces required to resist perturbations must increase linearly with the mass of the animal whereas the maximal force of a muscle scales with its cross-sectional area (and in turn this scales with mass according to $s_{CSA}=s_{m}^{2/3}$), more of the muscle must be activated in larger animals in order to resist the imposed perturbations. This observation is thus consistent with previous suggestions that the nervous system must play a more important role in maintaining joint configuration in larger animals [11,58].

Less straightforwardly, we also showed that the particular way that muscle activations scaled with body mass was strongly influenced by the presence of passive muscle forces and by the quadratic cost function used to resolve musculoskeletal redundancy when choosing muscle activations. These factors caused more muscles to be used to resist perturbations in larger animals, rather than simply increasing activation of the same set of muscles used by smaller animals. This observation trivially suggests that the same motor pattern used to produce behaviors such as locomotion in small animals cannot be identical to patterns used to produce similar behaviors in larger animals. As a consequence, this work reinforces the conclusions of previous studies that central pattern generators responsible for such behaviors must be adapted according to the specific mechanical properties of the animal, determined in part by the animal’s size [11,59,60,61,62].

Our simulations also suggested an interesting implication about how scaling influences the internal joint contact forces that determine joint integrity. We showed that the joint contact forces resulting from the muscle activations used to stabilize joint configuration increased with body mass with a scaling exponent of less than one. This was an unexpected result. As illustrated in Figure 4, inverse dynamic joint forces caused by the perturbations increased linearly with body mass whereas joint moments increased with the mass to the 4/3 power. In the simulation results illustrated in Figure 5, muscle activations were chosen to produce the net joint moments necessary to maintain joint configuration. Since those joint moments increased with the mass to the 4/3 power, we expected the joint contact forces to increase linearly with the mass. In fact, when we repeated the simulation without passive muscle forces and without a quadratic cost function, joint contact forces increased linearly with body mass, as expected. The sublinear increase in contact forces with body mass shown in Figure 5d thus likely reflected the wider distribution of activated muscles observed in larger animals.

One possible explanation for this result is that as more muscles are recruited, there is more co-activation between muscles producing opposing joint contact forces. For example, consider the activation of two muscles that both produce knee extension moments but that produce opposing tangential contact forces. If in animals with small body masses only one of these muscles has to be activated to produce the necessary joint moment, then only that muscle will contribute to the resulting joint tangential contact force. In larger animals, however, both muscles will be co-activated, resulting in a lower increase in the net tangential contact force than would be expected if only one muscle activation were increased. Thus, the control strategy used in these simulations, combined with the anatomy of the musculoskeletal system, might implicitly reduce joint contact forces in animals with increasing sizes, even though these contact forces were not explicitly controlled. This possible interaction between control strategies and musculoskeletal anatomy might be an interesting topic of future research, providing insights into the organization of musculoskeletal anatomy.

Of course, this work represents a limited consideration of the potential issues involved in the effects of body size on neural control. Limb anatomy, body posture, behavioral speed, or repertoire can all, in principle, be adapted according to overall body size [8,9,63,64]. Each of these factors will influence both the required properties of passive limb structures, such as the ACL, as well as the required role of the nervous system in responding to perturbations. How these factors interact with one another and why specific adaptations are observed in each animal, both in order to achieve task performance and in order to maintain joint integrity, will require a combination of experimental measurements and computational analyses, such as those described in this article.

## Data Availability

Data sharing not applicable.

## Abbreviations

ACL	Anterior cruciate ligament
CPG	Central pattern generator
CSA	Cross-sectional area
dof	Degree of freedom
